# Production of TNF-α, IL-12(p40) and IL-17 Can Discriminate between Active TB Disease and Latent Infection in a West African Cohort

**DOI:** 10.1371/journal.pone.0012365

**Published:** 2010-08-24

**Authors:** Jayne S. Sutherland, Bouke C. de Jong, David J. Jeffries, Ifedayo M. Adetifa, Martin O. C. Ota

**Affiliations:** Bacterial Diseases Programme, Medical Research Council Laboratories, Banjul, The Gambia; National Institute for Infectious Diseases L. Spallanzani, Italy

## Abstract

**Background:**

*Mycobacterium tuberculosis* (MTb) infects approximately 2 billion people world-wide resulting in almost 2 million deaths per year. Determining biomarkers that distinguish different stages of tuberculosis (TB) infection and disease will provide tools for more effective diagnosis and ultimately aid in the development of new vaccine candidates. The current diagnostic kits utilising production of IFN-γ in response to TB antigens can detect MTb infection but are unable to distinguish between infection and disease. The aim of this study was to assess if the use of a longer term assay and the analysis of multiple cytokines would enhance diagnosis of active TB in a TB-endemic population.

**Methods:**

We compared production of multiple cytokines (TNF-α, IFN-γ, IL-10, IL-12(p40), IL-13, IL-17 and IL-18) following long-term (7 days) stimulation of whole-blood with TB antigens (ESAT-6/CFP-10 (EC), PPD or TB10.4) from TB cases (n = 36) and their *Mycobacterium*-infected (TST+; n = 20) or uninfected (TST−; n = 19) household contacts (HHC).

**Results and Conclusions:**

We found that TNF-α production following EC stimulation and TNF-α and IL-12(p40) following TB10.4 stimulation were significantly higher from TB cases compared to TST+ HHC, while production of IFN-γ and IL-13 were significantly higher from TST+ compared to TST- HHC following PPD or EC stimulation. Combined analysis of TNF-α, IL-12(p40) and IL-17 following TB10.4 stimulation resulted in 85% correct classification into TB cases or TST+ HHC. 74% correct classification into TST+ or TST− HHC was achieved with IFN-γ alone following TB10.4 stimulation (69% following EC) and little enhancement was seen with additional cytokines. We also saw a tendency for TB cases infected with *M. africanum* to have increased TNF-α and IL-10 production compared to those infected with *M. tuberculosis*. Our results provide further insight into the pathogenesis of tuberculosis and may enhance the specificity of the currently available diagnostic tests, particularly for diagnosis of active TB.

## Introduction

Close to one-third of the world's population is infected with *Mycobacterium tuberculosis* (MTb), the causative agent of tuberculosis (TB), with infection rates highest in poverty-stricken countries throughout Africa and Asia [1]. Interestingly, while 8 million people develop active TB each year, the majority remain asymptomatically (latently) infected with the pathogen presumably due to a protective immune response. Determining what constitutes protective immunity to TB is critical for development of new diagnostics, treatment protocols and vaccine candidates. This requires the generation of an immune profile (or ‘biosignature’) corresponding to specific stages of TB infection.

IFN-γ is a cytokine produced by T cells that has been widely used for diagnosis of TB infection following stimulation with MTb-specific antigens [2]. However, IFN-γ alone is not sufficient to provide protection against disease progression [3], [4]. Other cytokines that have been found to be important in control of MTb infection include TNF-α [5], IL-12(p40) [6], IL-18 [7] and IL-17 [8]. For example, progression of LTBI to active disease can occur following TNF-α blocking treatments for chronic inflammatory diseases [5], while genetic defects involving IL-12 (as for IFN-γ [9]) increase susceptibility to development of active TB disease [6]. Increased Th2 cytokines (IL-4, IL-10) have been shown to be present in plasma of subjects with more advanced TB disease [10], while pro-inflammatory cytokines such as IL-6 and IP-10 are increased in subjects with active TB disease compared to those with LTBI [11]. In order to generate a biosignature for TB disease and infection a number of parameters need to be considered. These include the HIV status and age of the patient with the majority of diagnostic tests not applicable for either HIV-positive patients or children. Another potential confounder is the type of TB (pulmonary or extrapulmonary), the extent of disease and the sample type used for analysis. For instance, a decrease in IFN-γ production from peripheral blood cells has been shown to occur with advanced TB [12] but this may be due to sequestration of the cells at the site of infection [13].

The cytokine response to TB antigens has been studied extensively but results have varied due to the genetic background of the study population, the type and length of antigen stimulation, the experimental protocol used and the sample type. In addition, while most studies have shown preferential differentiation between infected and non-infected individuals, few have detailed differences between infection and active disease, essential for reducing TB transmission. One reason for this lack of differentiation may be due to the use of short term assays which will detect effector memory rather than generation of naive or central memory T cell responses [14]. Some studies using long-term stimulation have shown promising results including responses to Erp, an exported Mycobacterial protein [15] and to the 16kDa antigen Rv2031c [16], both of which have resulted in differential IFN-γ production in subjects with active disease compared to those with latent infection. Long-term assays have also been shown to enhance detection of LTBI and to distinguish between recently acquired and remote infections [17], [18].

In the present study, we used long-term (7 day) stimulation with PPD, ESAT-6/CFP-10 (EC) and TB10.4 followed by multiplex cytokine analysis of the supernatants. EC are secreted antigens encoded within the region of difference 1 (RD1) of the TB genome [19] and are also the basis of the currently available IFN-γ release assays (IGRA) such as the QuantiFERON-TB Gold [20]. TB10.4 is a recently identified antigen encoded by Rv0288 that has been found to be essential for the virulence of MTb [21] and thus may enhance discrimination between active TB and latent infection. Indeed, we found that combined analysis of TNF-α, IL-12(p40) and IL-17 production following stimulation with TB10.4 for 7 days resulted in 85% correct classification into subjects with active disease or those with latent infection. These results, once validated, may enhance the currently available cytokine-based TB diagnostic tests.

## Results

### Pattern of cytokine production following stimulation with PPD

**Figure 1:**
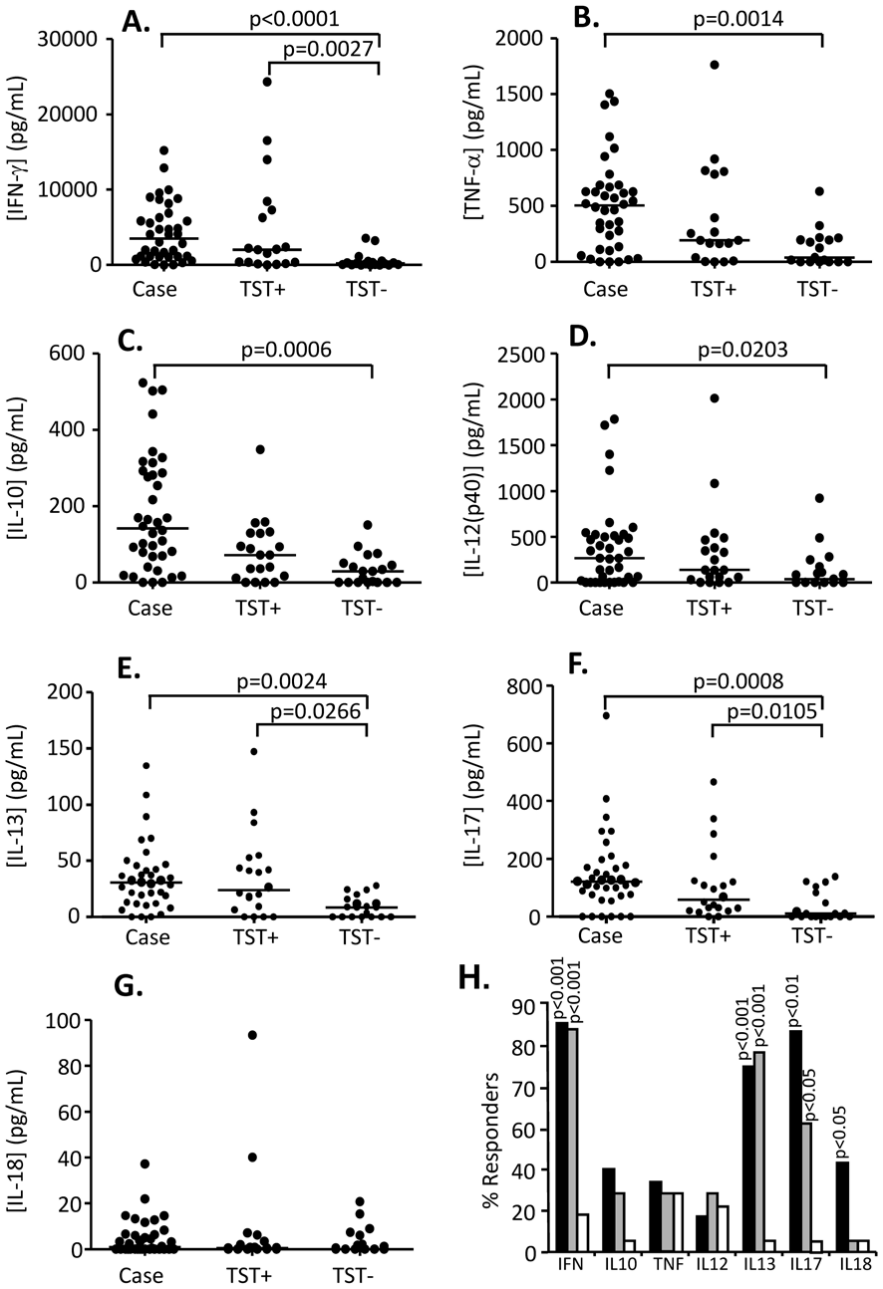
Cytokine responses following PPD stimulation. Whole blood from TB cases (n = 36), TST+ (n = 20) and TST− (n = 19) contacts was stimulated for 7 days with PPD and the supernatant assessed by multiplex cytokine analysis. A) IFN-γ. B) TNF-α, C) IL-10, D) IL-12(p40), E) IL-13, F) IL-17 and G) IL-18. Line indicates median. Data were analysed using Tweedie distributions with p-values indicating significant differences after adjustment for age and sex. H) Percentage of responders producing IFN-γ, IL-10, TNF-α, IL-12, IL-13, IL-17, IL-18. Dark bars = cases, grey bars = TST+ contacts, white bars = TST− contacts; p-values are indicated and refer to comparison with TST− contacts.

Following stimulation with PPD, production of all cytokines except IL-18 was significantly higher from TB cases compared to TST− contacts (Figure 1) while production of IFN-γ, IL-13 and IL-17 were all significantly higher in TST+ compared to TST− contacts (p = 0.0027; p = 0.0266 and p = 0.0105 respectively; Figs. 1A, E, F). No differences were seen between TB cases and TST+ contacts for any cytokine but TB cases had the highest level of all cytokines compared to TST+ and TST− HHC with IFN-γ being produced at the greatest amount (median[interquartile range (IQR)] = 2923[1101–5819]pg/mL for TB cases, 2043[316–7573]pg/mL for TST+ contacts but only 201[46–496]pg/mL for TST− contacts (Fig. 1A). TNF-α production (Fig. 1B) was also high from TB cases (median[IQR] = 476[117–628]pg/mL compared to 191[31–788]pg/mL for TST+ contacts and only 39[0–205]pg/mL for TST− contacts) with production of IL-13 low but still significantly higher than TST− HHC (30[12–45]pg/mL compared to 24[7–51] for TST+ and 8.5[0–16] for TST− HHC. Production of IL-18 was very low and was the only cytokine with no significant difference between any of the three groups (median[IQR] = 0.5[0–6.3] for TB cases, 0.5[0–3.1] for TST+ contacts and 0[0–6.1] for TST− contacts (Fig. 1G). Analysis of the proportion of responders was also performed as this further contributes to our understanding of the pathogenesis of TB. For each cytokine, responders were determined based on production >2SD above the mean level for the corresponding cytokine in the background sample. Following PPD stimulation there was a significantly higher proportion of TB cases and TST+ contacts producing IFN-γ, IL-13 and IL-17 compared to TST− contacts (p<0.0001 for all; Fig. 1H). Indeed, almost 90% of TB cases and TST+ contacts were responders based on IFN-γ production (compared to 20% of TST− contacts). Furthermore, almost 80% of TB cases and TST+ contacts responded on the basis of IL-13 production compared to <5% of TST− cases. Interestingly, while proportions were very low (<5% responders for both TST+ and TST− HHC), IL-18 showed a significant difference in the number of responders between TB cases and TST+ contacts (p<0.05, Fig 1H). The proportion of responders based on IL-17 production was also significantly different between TB cases (>80%) and TST+ contacts (60%; p<0.05; Fig. 1H).

### Pattern of cytokine production following stimulation with ESAT-6/CFP-10 (EC) fusion protein

**Figure 2:**
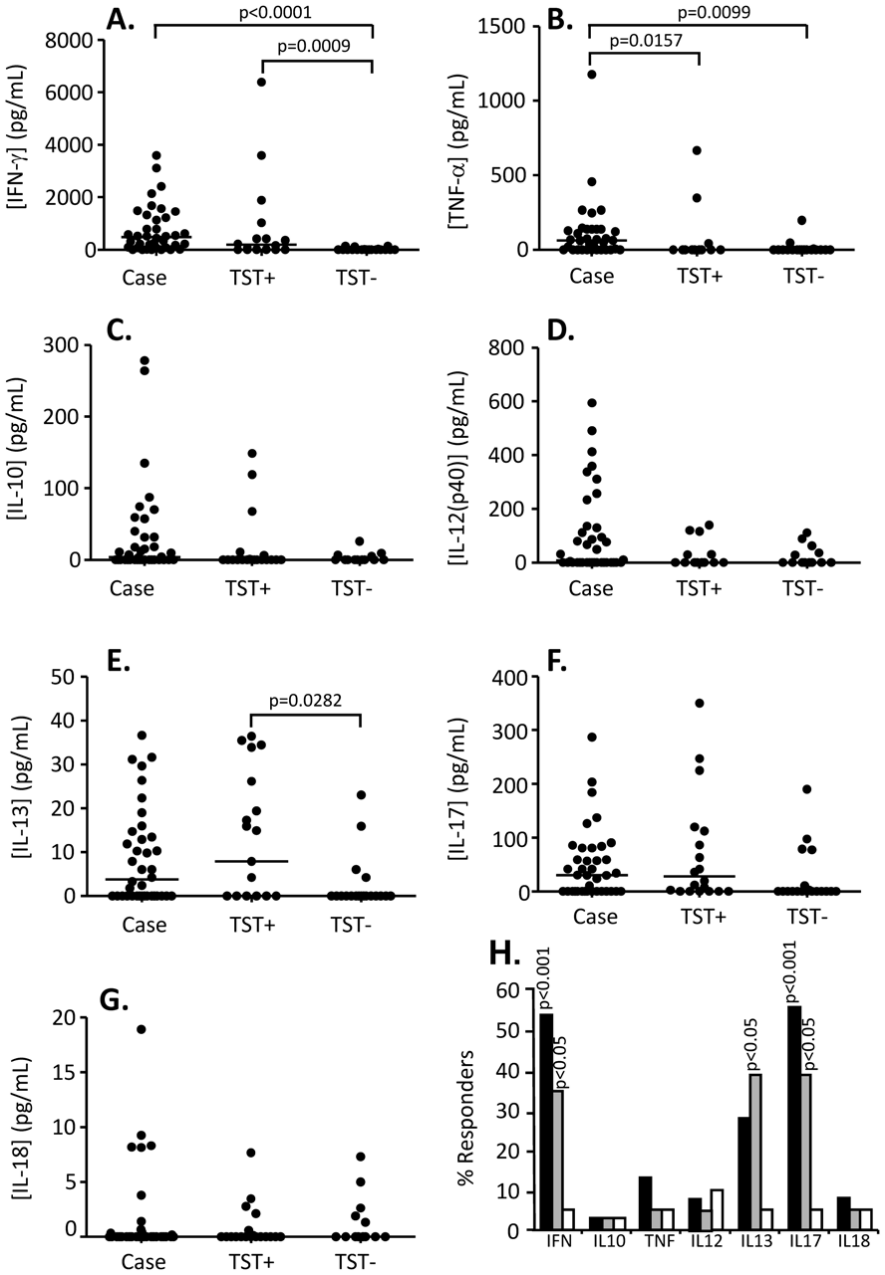
Cytokine responses following EC stimulation. Whole blood from TB cases (n = 36), TST+ (n = 20) and TST− (n = 19) contacts was stimulated for 7 days with ESAT-6/CFP-10 fusion protein and the supernatant assessed by multiplex cytokine analysis. A) IFN-γ. B) TNF-α, C) IL-10, D) IL-12(p40), E) IL-13, F) IL-17 and G) IL-18. Line indicates median. Data were analysed using Tweedie distributions with p-values indicating significant differences after adjustment for age and sex. H) Percentage of responders (prior to adjustment for age and sex) producing IFN-γ, IL-10, TNF-α, IL-12, IL-13, IL-17, IL-18. Dark bars = cases, grey bars = TST+ contacts, white bars = TST− contacts; p-values are indicated and refer to comparison with TST− contacts.

The responses seen following EC stimulation were similar to that following PPD stimulation but at a lower magnitude. For example, IFN-γ levels were (median[IQR]) 477[79–1303] pg/mL from TB cases and 198[0–1675] from TST+ contacts (compared to over 2000pg/mL following PPD stimulation) but was still significantly higher than for TST− HHC (0[0–19] pg/mL). IL-12(p40) and IL-17 levels were also high from TB cases but were virtually absent from both TST+ and TST− HHC, particularly for IL-12(p40) (median[IQR] = 5.1[0–14}, 0[0–30] and 0[0–28] for TB cases, TST+ and TST− contacts respectively). Production of IFN-γ and TNF-α were both significantly higher from TB cases compared to TST− contacts (p<0.0001 and p = 0.01 respectively; Figs. 2A and B) while TNF-α was significantly increased from TB cases compared to TST+ contacts (p = 0.0157; Fig. 2B). In addition TST+ contacts had significantly higher production of IFN-γ and IL-13 compared to TST− contacts (p = 0.0009 and p = 0.0282 respectively; Figs. 2A and E). The proportion of responders to EC was also lower than following PPD stimulation but the pattern of differences between the 3 groups was similar. A significantly higher proportion of TB cases and TST+ contacts produced both IFN-γ (p<0.001 and p<0.05 respectively) and IL-17 (p<0.001 and p<0.05 respectively) compared to TST− contacts (Fig. 2H). In addition, the proportion of TST+ contacts producing IL-13 was significantly higher than for TST− contacts (p<0.05; Fig. 2H). However, no difference in the proportion of responders was seen between TB cases and TST+ contacts for any cytokine. Only 55% of TB cases and 35% of TST+ contacts could be deemed to be responders based on IFN-γ production (compared to almost 90% following PPD stimulation), however, this was still significantly higher than TST− contacts with <5% producing IFN-γ at levels above background (mean+2SD).

### Pattern of cytokine production following stimulation with TB10.4

**Figure 3:**
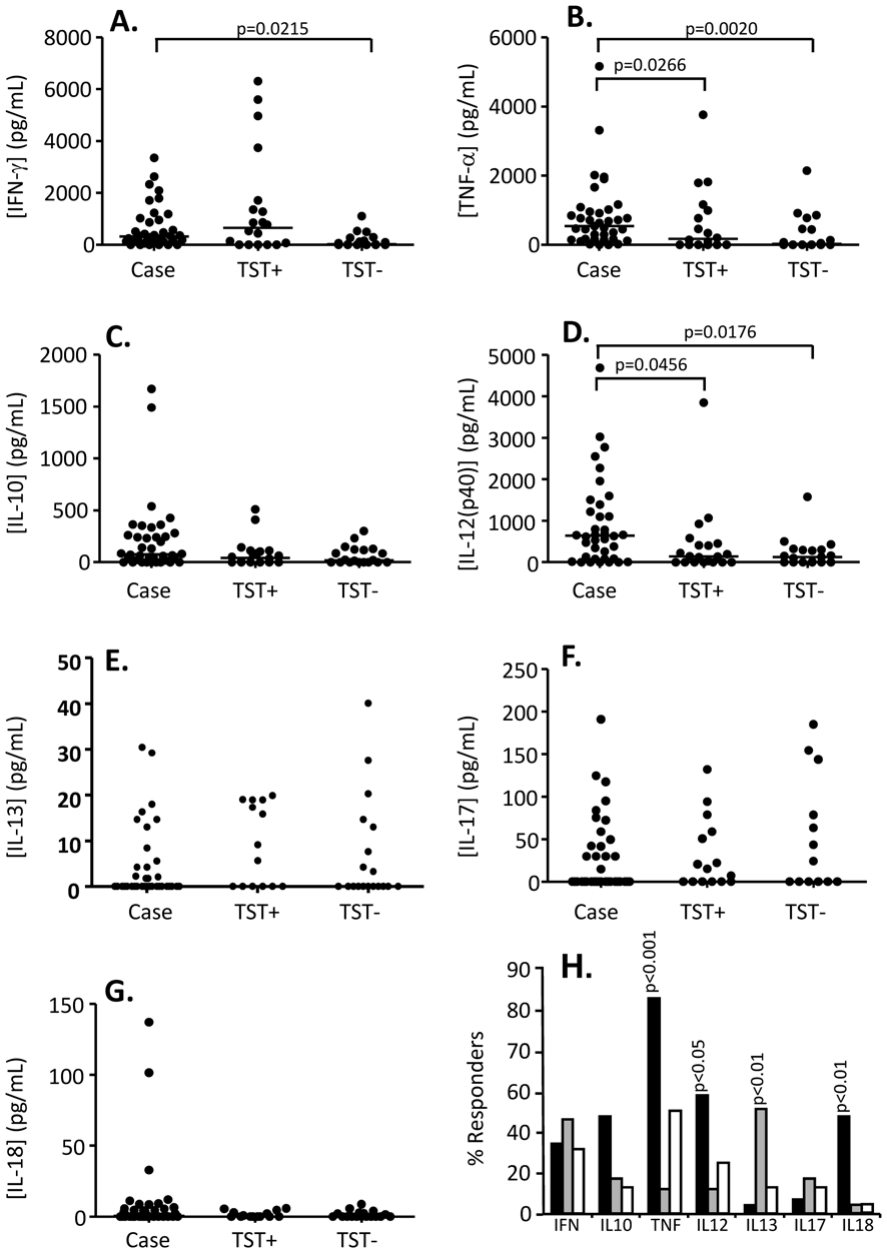
Cytokine responses following TB10.4 stimulation. Whole blood from TB cases (n = 36), TST+ (n = 20) and TST− (n = 19) contacts was stimulated for 7 days with TB10.4 and the supernatant assessed by luminex technology for secretion of cytokines. A) IFN-γ. B) TNF-α, C) IL-10, D) IL-12(p40), E) IL-13, F) IL-17 and G) IL-18. Line indicates median. Data were analysed using Tweedie distributions with p-values indicating significant differences after adjustment for age and sex. H) Percentage of responders (prior to adjustment for age and sex) producing IFN-γ, IL-10, TNF-α, IL-12, IL-13, IL-17, IL-18. Dark bars = cases, grey bars = TST+ contacts, white bars = TST− contacts; p-values are indicated and refer to comparison with TST− contacts.

Following stimulation with TB10.4, production of IFN-γ, TNF-α and IL-12(p40) were all significantly higher from TB cases compared to TST− contacts (p = 0.0215, p = 0.002 and p = 0.0176 respectively; Figs. 3A, B and D). No significant differences between TST+ and TST− contacts were seen for any cytokine but production of TNF-α and IL-12(p40) were both significantly higher from TB cases compared to TST+ contacts (p = 0.0266 and p = 0.0456 respectively; Figs. 3B and D). The production of these 2 cytokines was actually greater following TB10.4 stimulation than following PPD stimulation. Median[IQR] levels of TNF-α was 544[172–959], 175[0–1040] and 29[0–543] pg/mL respectively for TB cases, TST+ and TST− HHC (compared to 476[117–628]pg/mL for TB cases post-PPD stimulation) and for IL-12(p40) was 643[97–1476], 141[0–436] and 125[0–301]pg/mL for TB cases, TST+ and TST− HHC respectively (compared to 261[1.9–493]pg/mL for TB cases post-PPD stimulation). Analysis of the proportion of responders within each group showed significantly more TB cases producing TNF-α, IL-12(p40), IL-13 and IL-18 compared to TST+ contacts (p<0.001, p<0.05, p<0.01 and p<0.01 respectively; Fig. 3H) and TNF-α and IL-18 compared to TST− contacts (p<0.01 for both; Fig. 3H). No difference in the proportion of responders between TST+ and TST− contacts was observed. The pattern of production was different to that seen following PPD and EC stimulation. Whereas for both PPD and EC stimulation the most responders were seen with IFN-γ, IL-13 and IL-17 production, following TB10.4 stimulation the majority of responders was seen with TNF-α production (80% TB cases, 45% TST− HHC but <5% of TST+ HHC). Most other cytokines had less than 50% responders for any group.

### Multivariate analysis for classification of TB cases, TST+ or TST− contacts

We next used multivariate analysis to attempt to correctly classify subjects into TB cases, TST+ or TST− household contacts (Table 1). Following PPD stimulation, analysis of IFN-γ alone resulted in 74% correct classification into TB cases and TST+ HHC (Table 1). Combined analysis of IL-12(p40) and IL-13 resulted in lower sensitivity (89% compared to 94% for IFN-γ alone) but increased specificity (56%) resulting in 77% correct classification (Table 1). Analysis of TNF-α, IL-12(p40) and IL-13 together resulted in 81% correct classification; sensitivity was 94% and specificity was 56%. Following EC stimulation, analysis of IL-12(p40) alone resulted in 81% correct classification and there was a slight decrease (to 79%) with addition of other cytokines (Table 1). Following TB10.4 stimulation, analysis of IFN-γ alone resulted in 76% correct classification into TB cases or TST+ contacts while analysis of TNF-α, IL-12(p40) and IL-17 in combination increased this to 85% mainly due to an increase in specificity from 44% to 61% with sensitivity increasing from 92% to 97%. Thus our data shows enhanced classification if analysis is performed with multiple cytokines. ROC analysis determined a cut-off for active TB disease of TNF-α >166pg/mL, IL-12(p40) >210pg/mL and IL-17 >26pg/mL respectively.

**Table 1:** Multivariate analysis for classification into TB cases, TST+ or TST− contacts.

Antigen	Cytokines	% Sensitivity	% Specificity	% Correct classification
	**TB Cases versus TST+ HHC**			
**PPD**	IFN-γ	94	33	74
	IL-12(p40)+IL-13	89	56	77
	TNF-α+IL-12(p40)+IL-13	94	56	81
**EC**	IL-12(p40)	97	44	81
	IL-12(p40)+IL-10	91	50	79
	IL-12(p40)+TNF-α+IL-17	94	44	79
**TB10.4**	IFN-γ	92	44	76
	TNF-α+IL-12(p40)	97	56	83
	TNF-α+IL-12(p40)+IL-17	97	61	85
	**TST+ versus TST− HHC**			
**PPD**	IFN-γ	69	71	70
	IFN-γ+IL-10	69	77	73
	IFN-γ+IL-10+IL-12(p40)	69	77	73
**EC**	IFN-γ	72	61	67
	IFN-γ+IL-13	67	72	69
	IFN-γ+IL-17+IL-18	72	72	72
**TB10.4**	IFN-γ	83	65	74
	IFN-γ+IL-12(p40)	78	65	71
	IFN-γ+IL-12(p40)+IL-17	83	59	71

Multivariate analysis was also performed to attempt to classify TST+ and TST− HHC. We found that the level of % correct classification was lower for TST+ and TST− contacts than for differentiation between TB cases and TST+ contacts suggesting that the 7-day assay we used is better for discriminating between active disease and latent infection than between infection and non-infection, at least for the antigens we used. Following PPD stimulation, analysis of IFN-γ, TNF-α or IL-13 all resulted in 70% correct classification of TST+ or TST− HHC (Table 1; TNF-α, IL-13 not shown) with IFN-γ and IL-10 together giving 73% correct classification and this was not increased with addition of more cytokines (eg IFN-γ, IL-10 and IL-12(p40); Table 1). Following EC stimulation, IFN-γ alone resulted in only 67% correct classification, mainly due to low sensitivity, while analysis of IFN-γ together with IL-17 and IL-18 increased the percent correctly classified slightly to 72% (Table 1). Analysis of IFN-γ alone following TB10.4 stimulation gave the best classification into TST+ or TST− HHC (74% correct classification) and this was actually decreased with the addition of other cytokines (Table 1).

### Comparison of *M. africanum* versus *M. tuberculosis* infection

*M. africanum* (MA) is a common cause of TB in West Africa and is thought to be associated with an attenuated response to ESAT-6 [22]. As such we further analysed our results according to infection of cases with either MTb or MA (Table 2). IFN-γ production was highest for all stimuli regardless of infection type (median[IQR} for PPD = 2832[822–5679] pg/mL for MTb infection and 2495[1120–6082] pg/mL for MA infection; Table 3). There were no significant differences between the groups, however we did see a tendency for MA-infected cases to have increased TNF-α production (529[126–750] compared to 346[83–602]pg/mL; 115[0–137] compared to 44[0–122]pg/mL and 744[414–1924] compared to 455[154–937] pg/mL following PPD, EC or TB10.4 stimulation respectively; Table 3). In addition MA-infected cases had higher IL-10 production following PPD and TB10.4 stimulation (Table 3). IL-13, IL-17 and IL-18 production was similar regardless of infection type but IL-12(p40) showed variable results depending on the stimulation used (Table 3): following PPD stimulation levels were lower from MA- compared to MTb-infected cases (152[0–499] compared to 265[14–488] pg/mL) but higher following both EC and TB10.4 stimulation (74[0–373] compared to 0[0–103]pg/mL for EC and 939[80–2666] compared to 613[107–943]pg/mL for TB10.4; Table 3).

**Table 2:** Subject information.

	Case	TST− Contact	TST+ Contact
**n = **	36	19	20
**Age (median[IQR])**	25[20–37]	22[18–31]	27[19–39]
**Sex (% Male)**	73	26	35
**Genotype (MA∶MTb)**	14∶21	N/A	N/A

IQR = interquartile range; MA = *M. africanum*; MTb = *M. Tuberculosis*.

**Table 3:** Comparison of responses in *M. Africanum* versus *M. Tuberculosis* infected TB cases.

Stimulation	Group	n =	IFN-γ	TNF-α	IL-10	IL-12(p40)	IL-13	IL-17	IL-18
PPD	TB CASE	21	2832[822–5679]	346[83–602]	97[24–236]	265[14–488]	35[10–45]	118[28–172]	2[0–9]
	MA CASE	14	2495[1120–6082]	529[126–750]	152[62–301]	152[0–499]	28[13–49]	116[76–222]	0[0–6]
EC	TB CASE	21	440[130–1472]	44[0–122]	7[0–58]	0[0–103]	6[0–19]	57[0–82]	0[0–0.9]
	MA CASE	14	360[0–590]	115[0–137]	0[0–13]	74[0–373]	1.2[0–11]	0[0–48]	0[0–0.5]
TB10.4	TB CASE	21	338[120–496]	455[154–937]	73[0–237]	613[107–943]	0[0–5]	0[0–42]	1[0–9]
	MA CASE	14	252[0–1930]	744[414–1924]	163[22–406]	939[80–2666]	0[0–10]	0[0–80]	0.7[0–6]

TB = *M. tuberculosis*, MA = *M. africanum*. 0 = below limit of detection for assay; after background subtraction, negative values were converted to 0.

## Discussion

This study assessed cytokine production following 7-day stimulation of whole blood from TB cases and their latently infected (TST+) or non-infected (TST−) household contacts. Analysis of TNF-α, IL-12(p40) and IL-17 in combination following TB10.4 stimulation resulted in over 85% correct classification into active TB disease or latent infection. Analysis of IFN-γ resulted in 74% correct classification into TST+ or TST− HHC and additional cytokines did not enhance classification indicating that 7-day stimulation is optimal for discriminating between active disease and latent infection.

IFN-γ production following ESAT-6/CFP-10 stimulation is the basis of the currently available diagnostic tests for determining TB infection status but can't differentiate between infection and disease. The present study we found low sensitivity of IFN-γ production following EC stimulation for differentiating into TST+ or TST− HHC but good discrimination between infection and disease following TB10.4 stimulation indicating that a longer-term stimulation is optimal for detection of active disease. This may also be antigen-dependent since we only saw this with TB10.4 stimulation and, since TB10.4 is a virulent part of the MTb genome, it presumably plays an important role with increasing disease severity [21]. In this study we did not see any differences in cytokine production based on x-ray scores (data not shown) but these findings should be validated in a larger cohort where stratification according to x-ray scores, bacterial load and other epidemiological factors could be performed.

**Figure 4:**
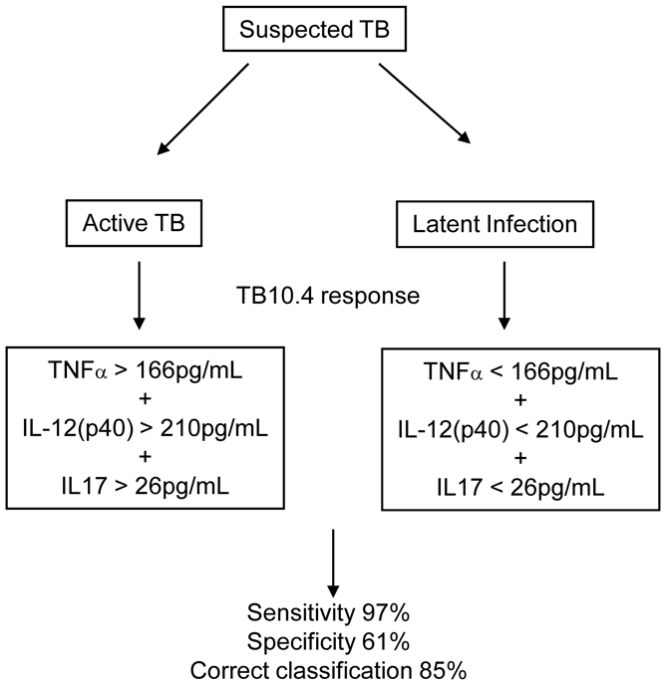
Flow chart demonstrating how cytokine production following TB10.4 stimulation will improve diagnosis of active TB. At the moment, patients with suspected TB may be confirmed by analysis of chest x-ray (CXR) plus sputum culture positivity in laboratories which have these capabilities. Analysis of TNF-α, IL-12(p40) and IL-17 production following TB10.4 stimulation should enhance classification into active disease or latent infection in laboratories without culture facilities and also for patients with suspected TB but smear/culture negative results (such as HIV-positive patients or extrapulmonary TB patients). These findings should be further validated in a short-term assay and in difficult to diagnose patient groups.

PPD stimulation resulted in differences in production of most cytokines between TB cases and TST− contacts but also a significantly higher level of IL-17 from TST+ compared to TST− contacts. IL-17 was also shown to enhance discrimination between TB cases and TST+ contacts following TB10.4 stimulation. Recent studies have suggested a role for IL-17 in protection against disease progression [8] with IFN-γ being shown to contribute to the regulation of the IL-17 producing cells [23]. We have previously shown that IL-17 is significantly decreased in TB cases compared to household contacts following overnight stimulation with both EC and PPD [24]. These differences presumably reflect the generation of a memory response to the TB antigens in the long-term culture compared to analysis of the immediate effector functions in overnight cultures. Therefore, in terms of development of a biosignature for active TB, our results suggest that long-term culture is required to distinguish between TB cases and TST+ contacts in a TB-endemic setting where the TST+ contacts have a relatively large pool of MTb-specific cells. The use of short-term assays will preferentially detect IFN-γ production by primed T cells [25], thus will show increased levels for both subjects with active disease and those individuals who have recently acquired infection thereby providing a potential explanation for lack of discrimination of active disease based on IFN-γ production following short-term antigenic stimulation. This is not to say that short-term assays won't be beneficial for detection of active disease in future, and indeed, are more clinically desirable than longer assays. Preliminary data from a recent study in South Africa did show differentiation between active disease and latent infection using short-term stimulation by analysing multiple cytokine/chemokine markers [26] but this will need to be validated in a larger cohort and at different sites in Africa due to genetic differences seen in response to TB antigens [27]. Our data shows that TB10.4 stimulation followed by multi-cytokine analysis allows discrimination between active disease and latent infection and should enhance classification of subjects, particularly those who are difficult to diagnose (ie HIV-positive or extrapulmonary TB) and at sites which lack x-ray and/or culture facilities (Fig. 4). Development of field-friendly, rapid diagnostics is essential in the fight against TB and analysis of cytokine responses following TB10.4 stimulation is promising; however it firstly needs to be validated in a short-term assay (Fig. 4). The relative use of peripheral blood IGRAs for diagnosis of active TB does appear to depend not only on the extent of pulmonary involvement but on the disease severity with low levels of cytokines (at least IFN-γ) present in more advanced disease [12]. While this may indicate less reactivity, it is more likely that the cytokine producing cells are simply required at the site of infection thus reducing the benefits of blood as a tool for diagnostics. Clearly more work is required but our results and others indicate the importance of generating an algorithm incorporating multiple factors that can accurately determine the TB status of an individual [28].

Due to the prevalence of *M. africanum* infection in The Gambia, we analysed whether this resulted in any differences in the host immune response. This is important since the progression from latent infection to active disease is increased in contacts of index cases infected with *M. tuberculosis* compared to *M. africanum* indicating a potential role for the bacteria in determining the outcome of the host immune response [29], [30]. However, while we saw a tendency towards increased IL-10 and TNF-α production in TB cases infected with MA compared to MTb, this had no significant effect on the overall cytokine profiles. Unfortunately we did not have enough contacts of index cases infected with either strain of bacteria to accurately assess the differences between them. Thus, while a similarity between MA and MTb in terms of the cytokine pattern is interesting, this should be validated in a larger sample size in order to provide a better platform for multivariate analyses.

In conclusion, this study shows that analysis of TNF-α, IL-12(p40) and IL-17 following long-term stimulation with TB10.4 results in 85% correct classification into TB cases and TST+ contacts and should be validated further for potential use in the next generation of immunodiagnostics.

## Methods

### Ethics statement

This study was conducted according to the principles expressed in the Declaration of Helsinki. Ethical approval was obtained from the Gambia Government/Medical Research Council Joint Ethics Committee. All patients provided written informed consent for the collection of samples and subsequent analysis.

### Participants

36 HIV-negative sputum smear-positive TB cases and 39 of their *Mycobacterium*-exposed household contacts were consecutively recruited as part of ongoing tuberculosis case-contact studies at the MRC (UK) unit in The Gambia and the Gates Grand Challenge 6, Biomarkers for TB. Subject information is presented in Table 2. Ethical approval was obtained from the joint Gambia Government/Medical Research Council Ethics committee and all subjects gave informed consent. All cases and contacts underwent a clinical assessment, including a screen for malaria and inter-current illness. Of the confirmed pTB cases, 2.5% had Grade I (+), 27.5% Grade 2 (++) and 70% Grade 3 (+++) smears (taken as highest grade out of 2 consecutively positive smears). Tuberculin skin tests (TST; 2 tuberculin units [TU], PPD RT23, SSI, Denmark) were performed in order to classify the household contacts into TST+ (n = 20) and TST− (n = 19). Due to the influence of prior BCG vaccination and environmental mycobacterial in this TB-endemic setting, subjects with skin test induration of ≥10mm diameter were categorised as TST positive. TB cases were further classified according to the degree of pulmonary involvement seen by chest x-ray into subjects with minimal disease (13/36; 36%), moderate disease (22%) and advanced disease (42%). Other clinical data such as night sweats, cough duration and weight loss were obtained from the TB cases where possible. Blood samples were taken from both TB cases and their contacts at recruitment. Of the TB cases, 35 had genotyped isolates, of which 14 (40%) were identified as *M. africanum* and the remainder as *M. tuberculosis*.

### Whole blood antigen stimulation

Blood was diluted 1∶10 in RPMI and added to wells containing antigen in triplicate. Antigens used were PPD (SSI; final concentration 10µg/mL), ESAT-6/CFP-10 fusion protein (EC; final concentration 10µg/mL) and TB10.4 (SSI; final concentration 10µg/mL). Unstimulated wells were used as a control for non-specific cytokine production. Plates were incubated at 37°C, 5% CO_2_ for 7 days. Approximately 90µl of culture supernatant was removed from triplicate wells, mixed in a single well in a new plate and stored at −20°C prior to multi-plex cytokine analysis.

### Multi-plex analysis for cytokine production

Culture supernatants were analysed using a Bio-Rad custom made 7-plex kit containing IL-10, IL-12(p40), IL-13, IL-17, IL-18, IFN-γ and TNF-α according to the manufacturer's instructions. Following pre-wetting of the filter plate, 50µl of bead suspension was added to each well and washed twice. 50µl of samples (tested singly) and standards (in duplicate) were then added, the plate sealed and shaken for 30 sec at 1100rpm then incubated for 1 hour at 300rpm. The plate was washed 3 times then 25µl of detection antibody added and the plate shaken and incubated for 30 min. at 300rpm. After washing, 50µl of 1× streptavidin-PE was added to each well and incubated for 10 min. The plate was again washed and resuspended in 125µl of assay buffer, sealed, mixed and immediately read on the Bio-plex analyser using Bioplex manager software (version 4.0) and a low PMT (photomultiplier tube) setting. The cytokine ranges were as follows (pg/mL). IL-10: 1.6–25201; IL-12(p40): 1.8–28172; IL-13: 0.3–4625; IL-17: 1.9–29871; IL-18: 2.1–31981; IFN-γ: 1.9–30205 and TNF-α: 5.9–92920.

### Mycobacteriology

Sputum samples were stained with auramine and the Ziehl-Nielsen (ZN) method. Sputum was decontaminated using N-acetyl cysteine (NALC)-NaOH before culture in Bactec vials (Becton Dickinson) and on paired Lowenstein-Jensen slopes. Positive cultures were confirmed by ZN smear and stored in glycerol at −70°C.

### Mycobacterial genotyping

Stored isolates were grown in Middlebrook 7H9 broth with OADC (oleic acid, albumin, dextrose, and catalase) supplement for DNA extraction. 10ng of DNA was used for spoligotype analysis with commercially available membranes (Isogen Biosciences, The Netherlands). Spoligotype films were scanned and classified using software designed in Matlab (Mathworks, USA), followed by manual editing and confirmation. Each spoligotype pattern was classified into a binary code and the result was entered in a Microsoft Access database (Redmond, USA). For isolates that could not be reliably classified as *M. tuberculosis* or *M. africanum* based on spoligotype analysis alone, the presence or absence of lineage defining large sequence polymorphisms RD702 and TbD1 was assessed, as previously described [30].

### Statistical analysis

Antigen-specific samples had background subtracted (unstimulated results) and negative values were adjusted to zero. The 3 groups of subjects were compared using generalised linear regression assuming a Tweedie distribution, which allows the zero inflation (caused by non-responders) to be modeled [31]. This allows adjustment for age and sex and results in greater power than can be achieved using standard non-parametric tests. Univariate logistic regression was also performed. Significance was tested at 5% and quoted p-values are adjusted for multiplicity using Bonferroni correction. For each cytokine, responders were defined as those producing levels >2SD from the mean of the unstimulated samples for each group (cases, TST+ and TST−). Multivariate discrimination was tested using a support vector machine [32], with testing and training sets selected using holdout cross-validation with half the data allocated to each training set. ROC analysis was also performed to determine cut-off levels and likelihood ratios for each analyte. *M. africanum* versus *M. tuberculosis* results were analysed using Mann-Whitney U tests. All analyses were performed following background subtraction of the samples using STATA version 9.1 (Stata Corporation, USA) and Matlab version 7.6 (Mathworks, Natwick, 2008).
